# Binding mode information improves fragment docking

**DOI:** 10.1186/s13321-019-0346-7

**Published:** 2019-03-22

**Authors:** Célien Jacquemard, Malgorzata N. Drwal, Jérémy Desaphy, Esther Kellenberger

**Affiliations:** 1 Laboratoire d’innovation thérapeutique, UMR7200, CNRS, Université de Strasbourg, 67400 Illkirch, France; 2 Lilly Research Laboratories, Eli Lilly and Company, Indianapolis, IN 46285 USA

**Keywords:** Ligand/protein complex, Fragment-based drug design (FBDD), Docking pose, Scoring

## Abstract

Docking is commonly used in drug discovery to predict how ligand binds to protein target. Best programs are generally able to generate a correct solution, yet often fail to identify it. In the case of drug-like molecules, the correct and incorrect poses can be sorted by similarity to the crystallographic structure of the protein in complex with reference ligands. Fragments are particularly sensitive to scoring problems because they are weak ligands which form few interactions with protein. In the present study, we assessed the utility of binding mode information in fragment pose prediction. We compared three approaches: interaction fingerprints, 3D-matching of interaction patterns and 3D-matching of shapes. We prepared a test set composed of high-quality structures of the Protein Data Bank. We generated and evaluated the docking poses of 586 fragment/protein complexes. We observed that the best approach is twice as accurate as the native scoring function, and that post-processing is less effective for smaller fragments. Interestingly, fragments and drug-like molecules both proved to be useful references. In the discussion, we suggest the best conditions for a successful pose prediction with the three approaches.

**Electronic supplementary material:**

The online version of this article (10.1186/s13321-019-0346-7) contains supplementary material, which is available to authorized users.

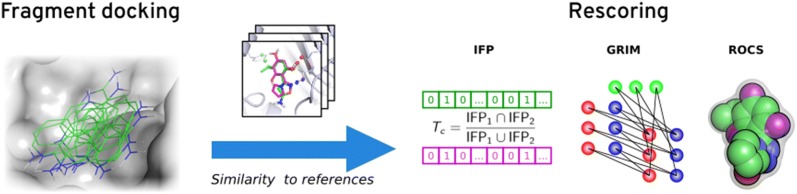

## Introduction

Fragment-based screening approaches have emerged as effective and complementary alternatives to high throughput screening (HTS), opening new avenues for drug design [1]. A recent survey of fragment literature has outlined the growing interplay between industry and academia as well as between pharmaceutical sciences, chemistry, biology, physics and computing [2]. Computational approaches have a special place, as they have been pioneers in the mapping of sites by very small molecules [3, 4]. Methods developed to predict binding of a ligand to a target protein constitute a cost-effective way to virtually screen large chemical libraries. In addition they are not limited to the previously synthesized molecules, thus presenting the advantage of enabling the screening of new chemotypes [5].

Molecular docking is a method of choice for the search for original hit compounds. For example, in a discovery effort of the A2A adenosine receptor, virtual screening provided new fragments although many ligands were already reported for this target protein [6]. Other recent successful fragment-based drug discovery programs showed the successful contribution of docking to the design of fragment inhibitors of enzymes [7, 8]. Docking can also assist the growing of fragment hits by predicting binding pose of the proposed compounds [9–11]. An accurate binding pose model is of prime importance to these two applications of the docking method.

Molecular docking can be thought of two separate but related phases. First is the “sampling” phase where the 3D pose of the ligand into the protein receptor is explored. Typically, many hundreds or thousands of potential poses are sampled. Phase two is the “scoring” phase in which a scoring function is used to order the sampled poses and ultimately produce the top set of predicted poses and their scores. These phases are interrelated, since the scoring function is also used to drive the sampling, but can be thought of as two separate problems. Docking programs typically generate multiple possible ligand poses with an associated score, but identifying the correct binding pose out of the set of possibilities is still an issue. In a recent example of fragment-based drug discovery, docking poses used to guide the design of PIM-1 kinase inhibitors have not been validated by X-ray crystallography (the predicted polar interactions were correct, but hydrophobic different contacts were different) [12].

Benchmarking studies have demonstrated that the use of experimental information on binding mode improves pose prediction of drug-like ligands [13–18]. Recent docking challenges revealed that experimental 3D-structures of ligand–protein complexes are widely used to re-rank docking solutions [19, 20]. For example, the participants of Drug Design Data Resource (D3R) Grand Challenge had to blindly predict the conformation of 36 drug-like ligands bound the farnesoid X receptor. Several participants scored poses by similarity to reference 3D-structures which were 3D-aligned based on shape, pharmacophoric features, or the interactions made between the ligand and the protein. Half of them made overall good predictions, with an average RMSD computed between the native and predicted poses of ~ 3 Å.

Scoring by similarity is fast but requires the 3D-structures of reference complexes. The power of the approach depends on the coverage of protein interactions by the reference molecules, and therefore it is desirable that the ensemble of reference molecules provides a comprehensive description of interactions made by the protein. Our recent analysis of the Protein Data Bank (PDB) suggested that fully mapping a pocket is achieved by nine different fragments or nine different drug-like ligands [21].

**Fig. 1:**
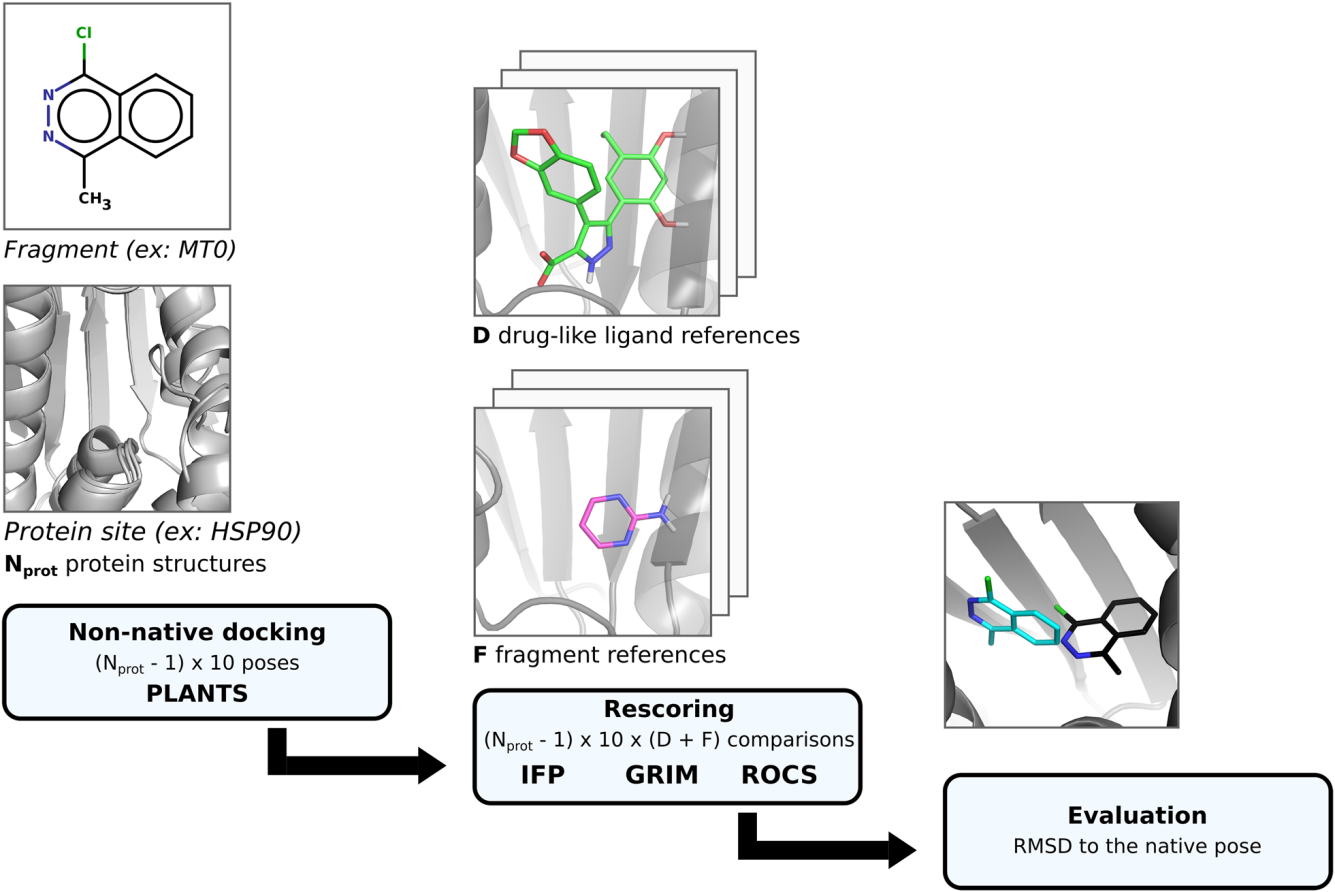
General protocol of pose prediction and its evaluation

Here, we explore the rescoring performance on fragment pose prediction of three rescoring approaches based on the 3D-structure of reference complexes: similarity of interaction fingerprints (IFP) [22], graph matching of interaction patterns (GRIM) [23] and rapid overlay of chemical structures (ROCS) [24] according to shape and pharmacophoric properties. We searched the PDB for proteins crystallized with both fragments and ligands. For every fragment, we performed all possible cross-dockings into its target protein site. Poses were rescored using structural information on all the other fragments and drug-like ligands of this protein site. Solutions were evaluated by considering the deviation to the native pose (Fig. 1). In analysing the benchmarking results, we aim to answer the following questions:

- Do the three methods have comparable performance levels (as measured by the deviation to the crystallographic structure coordinates)?
- Are the fragments more appropriate references than drug-like ligands?
- Are molecules chemically similar to the docked fragment better references?

## Experimental section

### Selection of PDB files

We defined a fragment as an organic molecule which is small but not a crystallization additive (such as buffer or precipitant). Size selection rules were a molecular weight (MW) below 300 Da and a number of non-hydrogen atoms between 2 and 18. We looked for drug-like ligands in the sc-PDB [25], keeping only those which follow the Rule of 5 [26] with up to one exception and which are heavier than fragments (MW > 300 Da). In a previous exploration of the publicly available data from the RCSB PDB web site, we retrieved 235 proteins in complex with at least one fragment and one drug-like ligand [21]. Only high quality 3D-structures were considered: resolution ≤ 3 Å; deposition date > 2000 and < 2016; no mutated, incomplete or missing residues in the protein binding site; no incomplete ligands; good fit of electron density map to the ligand and protein site structures using EDIAscorer v1.0 (median EDIA ≥ 0.8) [27]. In this study, we retained the proteins described by at least 3 PDB files representing three different complexes involving the same site.

### Protein and ligand preparation

3D-structures were downloaded from the RCSB PDB web site [28] and prepared as previously described [21]. The complexes were automatically protonated using Protoss v2.0 [29]. Importantly, water or cofactor molecules were not preserved in the protein site. In addition, all structures of the same protein were 3D-aligned to a reference structure using CE [30]. The reference protein structure was chosen as the centroid according to binding site similarity. Proteins and small molecules were saved in separate MOL2 files. A binding site includes all residues having at least one atom at less than 6.5 Å around the bound ligands. Here, we considered a consensus site where each residue is present in the binding site of at least 10% of its PDB complexes (more details in [21]).

If multiple structures were available for the same fragment within the same protein site (if the PDB file contains several biounits or if the same complex is described in two PDB files), different bound conformations were picked according to the root mean square deviation (RMSD) of the non-hydrogen atoms coordinates. In detail, duplicate fragments were identified by comparing canonical SMILES strings generated using the OpenEye Python2.7 API version 2017.Oct.1 (OpenEye Scientific Software, Santa Fe, NM. http://www.eyesopen.com). RMSD values were computed on non-hydrogen atom coordinates using Surflex-dock v3066 [31]. Conformations were distinguished using a hierarchical clustering (average linkage) based on the RMSD values with a 0.5 Å cut-off. The same clustering procedure was applied to drug-like ligands to keep only diverse conformations of a drug-like ligand within a protein site. Importantly, a single conformation was used as docking input. It was chosen as the most representative structure (i.e., the cluster center).

### Docking of fragments

Docking was performed with the PLANTS v1.2 program using the ChemPLP scoring function and the search speed 1 (highest accuracy) [32]. PLANTS is based on an ant colony algorithm to optimize the placement and the conformation of ligand as well as the positions of the protein hydrogen atoms that form hydrogen bonds with the ligand. PLANTS explores possible torsion angle values of the ligand but does not modify the conformation of rings.

The cavity center of a protein site was defined from the centroid of all the fragments and drug-like ligands bound to this protein. The cavity radius was set as the maximum distance between the cavity center and the atoms of all the ligands crystallized in the binding site (fragments and ligands), plus 2 Å. On average, the radius was equal to 11.2 Å. Ten poses were saved per docking run.

The input conformation of docked fragment came from the crystal structure of a parent complex (see the above paragraph for the selection of coordinates when the fragment is present in more than one complexes). Of note, PLANTS “sampling” performances were not changed if fragment structures were generated ab initio (Additional file 1: Figure S1). The docking of a fragment into its protein site was repeated in all the structures of this protein (≥ 3 structures per protein site, see the above mentioned selection rules).

### Rescoring

Each docking pose was then rescored using the IFP, GRIM and ROCS methods which are described below.

**Fig. 2:**
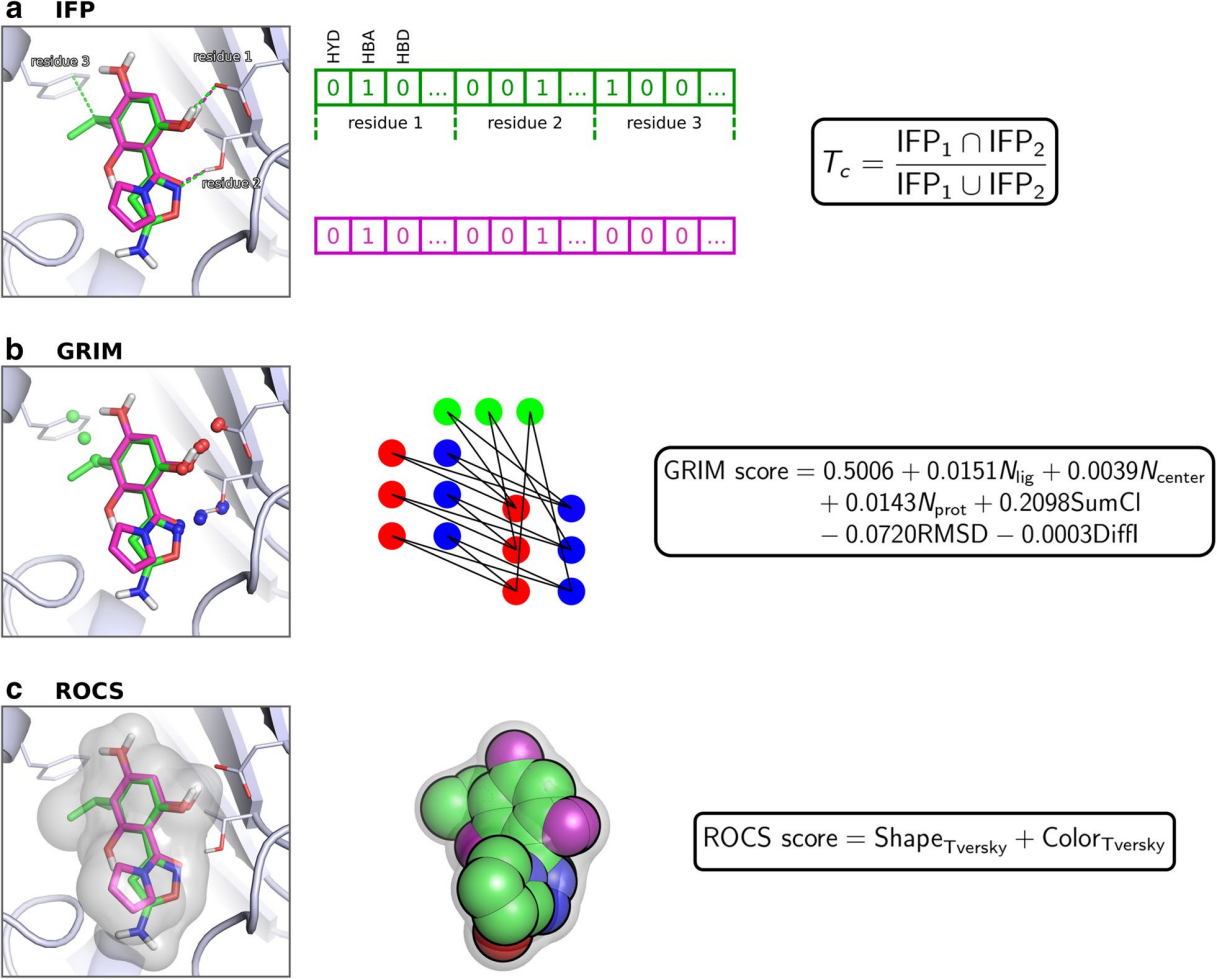
Overview of the rescoring methods. IFP *Tc* denotes Tanimoto coefficient. In the GRIM score *N*_*lig*_ is the number of aligned ligand points, *N*_*center*_ the number of aligned centered points, *N*_*prot*_ the number of aligned protein points, *SumCl* the sum of clique weights over all weights, *RMSD* the root-mean square deviation of the matched clique and *DiffI* the difference between the number of interaction points in the query and the reference. ROCS score is based on Tversky coefficient

IFPs are bitstrings which encode the binding mode of a ligand to its protein site (Fig. 2a). Every site residue defines a substring of the fingerprint where each bit represents a different interaction type (hydrophobic contact, hydrogen bond, ionic bond, face-to-face π stacking, face-to-edge π stacking, π-cation, and metal interaction) and “1” means that the interaction is detected between the ligand and the residue. Substrings are ordered according to site residues numbering. If a protein contains one or more metal cofactors, we consider as many metal residues as different coordination spheres in the crystallographic structures of the protein. The similarity between two IFPs is evaluated with the Tanimoto coefficient. In this study, we ensured that polar interactions contribute to similarity by nullifying it if the Tanimoto coefficient computed on polar interactions only was < 0.2. IFPs were generated using IChem v5.2.9 with an extended representation (-extended) and the maximum π–π interaction threshold set to 5.0 Å.

In the GRIM approach, the binding mode of a ligand to its protein is encoded into Interaction Pseudo Atoms (IPA, Fig. 2b). Each interaction is represented by a triplet of IPA: the first is located on the protein atom, the second on the ligand atom and the third in the middle of the interaction. IPA are labelled by interaction type (hydrophobic contact, hydrogen bond, ionic bond, face-to-face π stacking, face-to-edge π stacking and metal interaction). The similarity between two IPA maps is deduced from their graph alignment matching (only identical IPAs are paired). In this study, we checked that at least four IPA pairs, including at least a polar one, superimpose. If the condition was not fulfilled, the similarity was nullified. IPA were generated using the *ints* module in IChem v5.2.9. All hydrophobic points were considered (-noMerge option) and the maximum π–π interaction distance was set to 5.0 Å. The similarity was computed using the *grim* module of IChem v5.2.9 (default settings).

Last, docking poses were rescored using ROCS v3.2.0.4 (OpenEye Scientific Software, Santa Fe, NM. http://www.eyesopen.com), which evaluates the overlap of shape and pharmacophore features (-scoreonly option) (Fig. 2c). The pharmacophore features are described in the Implicit Mills Dean color force field files and include hydrogen-bond donors, hydrogen-bond acceptors, anions, cations and hydrophobic groups. Similarity was measured using the Tversky combo score, with α = 0.95 on the docking pose and β = 0.05 on the reference crystal structure.

## Results

### Description of the benchmark set

**Fig. 3:**
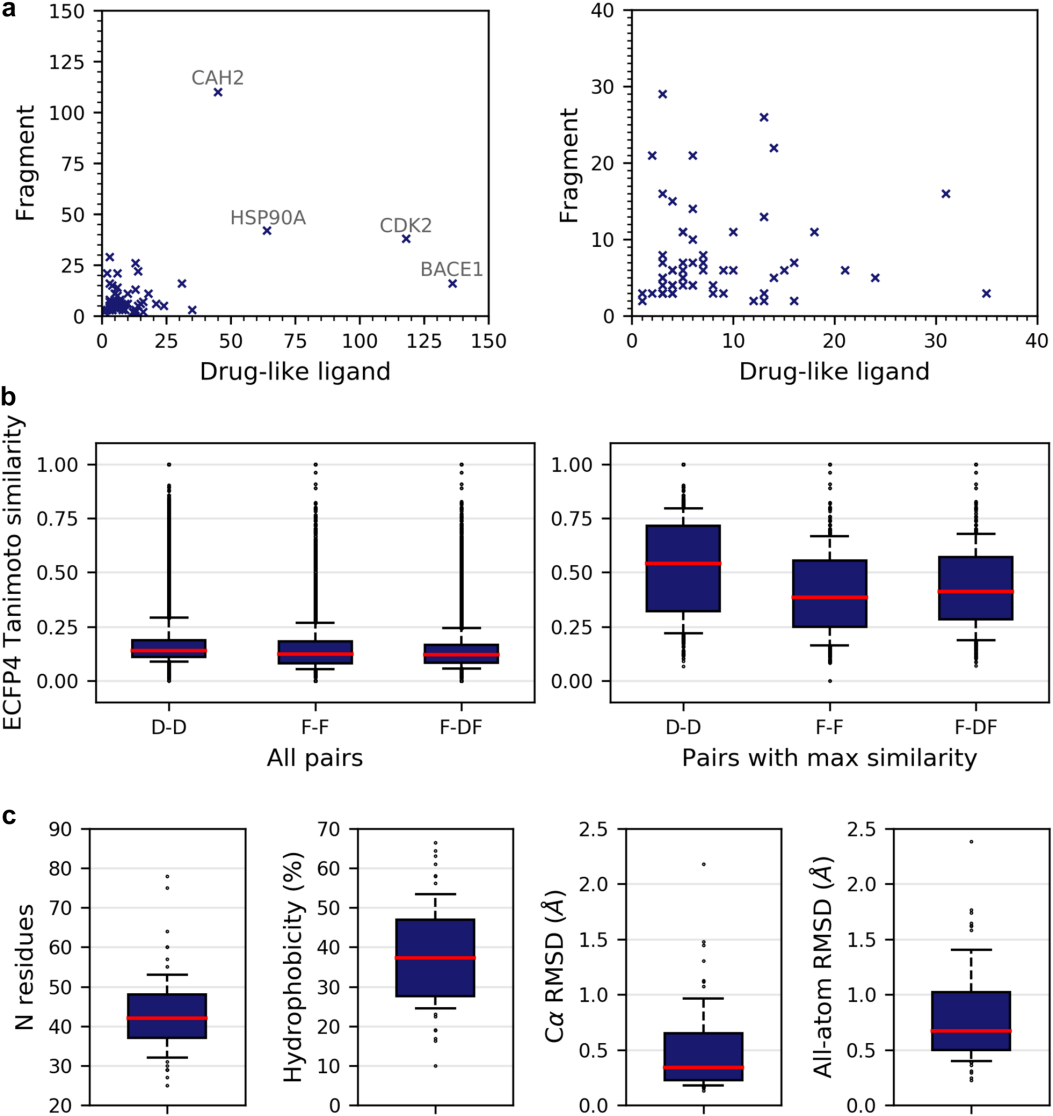
Description of the dataset. **a** Number of fragments and drug-like ligands HET codes per protein. The figure on the right zooms to the most populated area of the figure on the lower-left. **b** Molecular diversity of protein ligands. For every protein, ligands are compared with each other (D–D), fragments are compared to each other (F–F) and fragments are compared to drug-like ligands and fragments (F-DF). The distribution of similarity values is given for all pairs (left) and considering the maximal value only (right). **c** Properties of protein sites. From left to right: number of residues (*N residues*), relative hydrophobicity (*Hydrophobicity*), structure variations in the backbone (*Cα RMSD*) and structure variations in the backbone and the side chains (*All atom RMSD*). Distributions are shown for the complete set of structures. Boxplot whiskers represent the 1st and the 9th deciles

We selected from the PDB 2376 high-quality structures representing 64 proteins which accommodate both fragments and drug-like ligands within the same ligandable cavity. On average, there are 10.3 fragments (2 to 110 HET codes) and 13.3 drug-like ligands (1 to 136 by HET codes) per protein (Fig. 3a, Additional file 1: Table S1). A total of four proteins have been crystallized with more than 50 different small molecules. Cyclin-dependent kinase (P24941), Carbonic Anhydrase (P00918), Beta-secretase (P56817) and Heat shock protein HSP 90-alpha (P07900) show the highest number of molecules (156, 155, 152 and 106 respectively).

For most of the proteins, the fragments constitute a diverse set of chemical structures. The median Tanimoto index computed on ECFP4 fingerprints is lower than 0.3 for 93% of fragments pairs (Fig. 3b). The similarity between the fragments and drug-like ligands is also low (median ECFP4 Tc < 0.3 for 90.6% of pairs). Nevertheless, 18% of the fragments are highly similar to another one (max ECFP4 Tc > 0.6). This proportion increases to 20% when considering fragment/drug-like pairs.

The 64 proteins cover a wide range of activities with 18 transferases, 16 hydrolases, 10 oxidoreductases, three ligases, two receptors, two ion channels, two isomerases, two activators, one chaperone, one chromatin regulator, one toxin, one signal transduction inhibitor, one lyase and four miscellaneous proteins (Additional file 1: Table S1). Binding sites are of various size and composition (Fig. 3c). The number of residues ranges from 25 for the smallest site in the bromodomain-containing protein 4 to 78 for the largest site in the β-1 adrenergic receptor. The majority of sites expose both hydrophobic and polar groups to the protein surface (median hydrophobicity equal to 36%). The most hydrophobic site is found in the oestrogen receptor β, whereas the most polar site is found in the methionine aminopeptidase.

Binding sites are mostly rigid (Fig. 3c). The RMSD computed on all non-hydrogen atoms of the amino acids in the binding site is lower than 1.0 Å in about three quarters of the 3D-structures pairs. Only one site shows an important variation of the backbone conformation. This is the metallothionein-2 with a maximal RMSD computed on Cα atoms of 2.4 Å. For the sake of comparison, the second most flexible protein in the set (the ketohexokinase) shows a maximal RMSD computed on Cα atoms of 1.5 Å (1.7 Å if all non-hydrogen atoms are considered).

### Quality and diversity of the docking poses

**Fig. 4:**
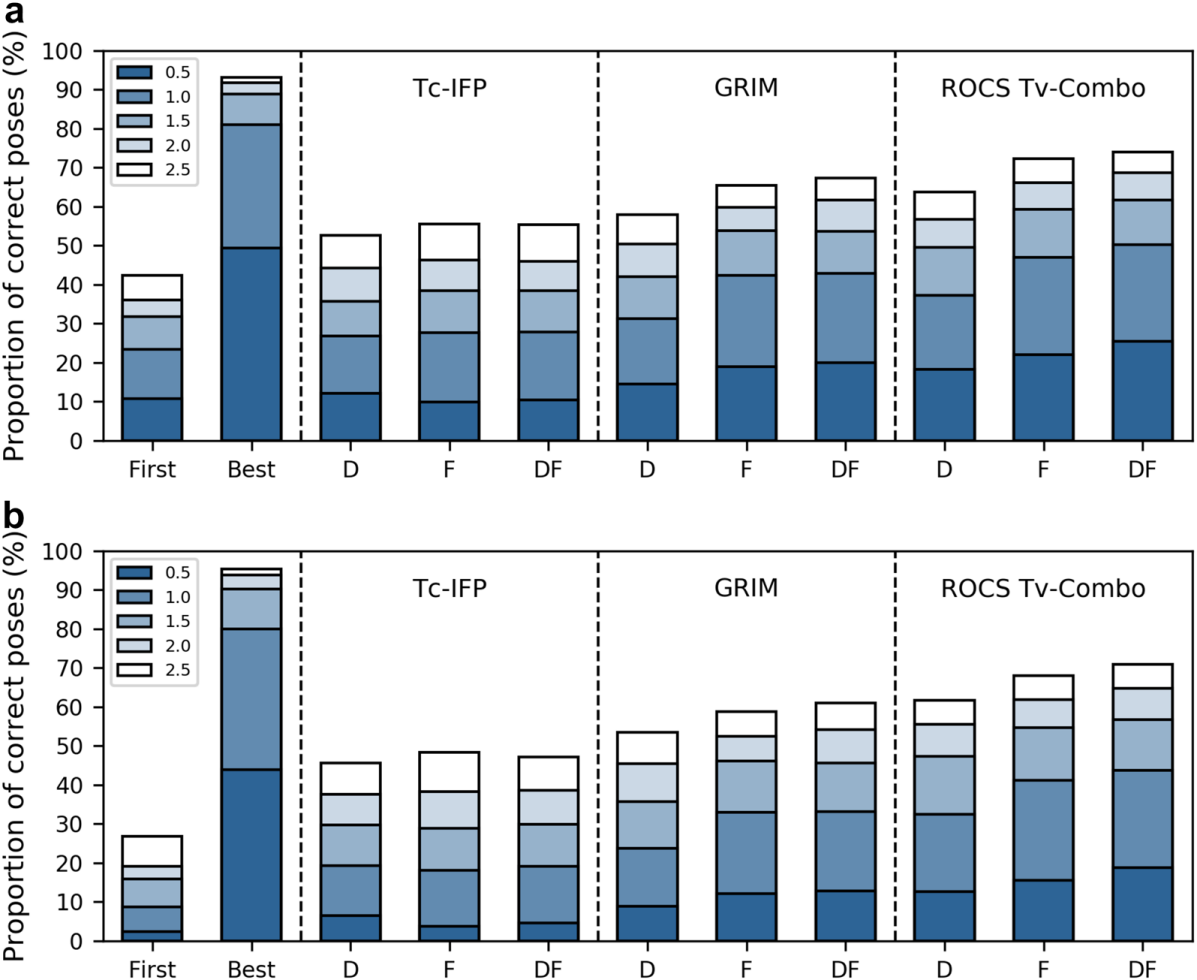
IFP, GRIM and ROCS performance in pose prediction. The proportion of correct predictions is based on the RMSD between the predicted and native poses of fragment, considering five threshold values. Proportions are calculated by considering a single pose within the ensemble generated for a complex, as follows: *First* denotes the top scored pose; *Best* denotes the closest to the native pose; *D*, *F* and *DF* denote the poses selected by comparison to, respectively, reference drug-like ligands, reference fragments and both. **a** All protein sites. **b** The 35 protein sites with frequent scoring issues (scenario 3)

For all the 586 fragment/protein complexes, the crystallographic structure of the fragment was docked into all the structures of the protein except that of the native crystal complex (non-native or cross-docking). The number of poses generated for a fragment ranges from 20 to 1400 depending on the number of protein site structures. In about one third of the fragment/protein complexes studied, a docking solution close to the native pose is ranked first by the ChemPLP scoring function (*First* pose in Fig. 4a). More precisely, in 64% of complexes, the RMSD between the docked and native poses is above the 2 Å threshold which is commonly used to evaluate docking accuracy. Nevertheless, a correct docking solution is found in almost all ensembles of poses (see *Best* pose in Fig. 4a), indicating that the problem is not the “sampling” phase of the docking, it is the “scoring” phase. Good poses are being produced, but they are not being identified by the scoring function. We distinguished three rescoring scenarios. In the first one, the scoring function generally selects a correct solution and thus rescoring is useless. In the second one, by contrast, most of the docking solutions are wrong and thus rescoring exercise is hardly possible. The third scenario corresponds to the most interesting cases, where the correct docking pose is predicted in the ensemble of poses (20 to 1400, depending on the number of protein input structures), but it is not the top scored pose. We defined that a protein site experiences the first scenario if the scoring function retrieves a correct top scored pose for 50% or more of the fragments and that it experiences the second scenario if 50% or less of the fragments show a minimal RMSD < 2 Å. About one half of the proteins do not meet these two definitions. These 35 proteins correspond to 389 fragments. Their docking yielded scoring issues in about 80% of the pose predictions (Compare *First* and *Best* in Fig. 4b). By comparison, in the 24 proteins in scenario 1, the native scoring function ChemPLP retrieves a correct top scored pose for more than 80% of the pose predictions (Additional file 1: Figure S2A). Conversely, virtually no correct poses are selected for the 5 proteins in scenario 2, where docking failures predominate (no correct solutions at all for a majority of fragments, Additional file 1: Figure S2B).

### Rescoring with IFP, GRIM and ROCS

#### Comparative evaluation of the methods

The three rescoring methods improved the pose prediction of fragments, however, with variations in the level of improvement (Fig. 4). On the whole dataset, IFP performs better than ChemPLP in pose ranking. When considering the top ChemPLP score, the RMSD between the docked pose and the native pose is lower than 2 Å in 37% of docking experiments. This value reaches 44 to 46% if the docked pose is selected based on IFP Tc rank. IFP shows better performance on the 35 proteins with frequent scoring issues (scenario 3). In those cases, the proportion of good poses selected by IFP is twice higher than that selected by ChemPLP. By contrast, IFP deteriorates fragment pose prediction in the 24 proteins of scenario 1 (Additional file 1: Figure S2A). GRIM is more efficient than IFP on both the entire set and the 35 proteins with frequent scoring issues, by enabling the retrieval of good poses in 9% to 18% additional cases, depending on the reference molecule type (see below). In addition, GRIM is able to perform as well as ChemPLP in scenario 1. The best results are obtained using ROCS, which yields a success rate exceeding 60% on the entire dataset. Interestingly, the same success rate is obtained with the combo-Tc and combo-Tv scores, suggesting that ROCS rescoring performance is not affected by size differences between the docked and the reference molecules (Additional file 1: Figure S3). By contrast, IFP rescoring performance is slightly decreased when the Tversky coefficient is used instead of the Tanimoto coefficient (Additional file 1: Figure S3).

#### Reference molecules type

**Fig. 5:**
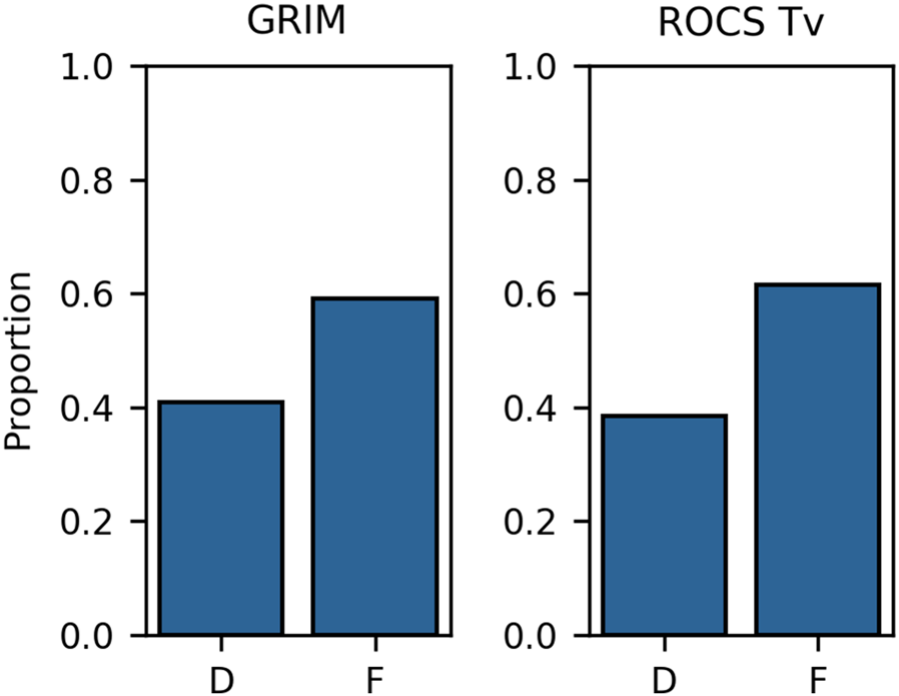
Type of reference molecules picked by GRIM and ROCS Tv-combo. *D* and *F* denote drug-like ligand and fragment, respectively

**Fig. 6:**
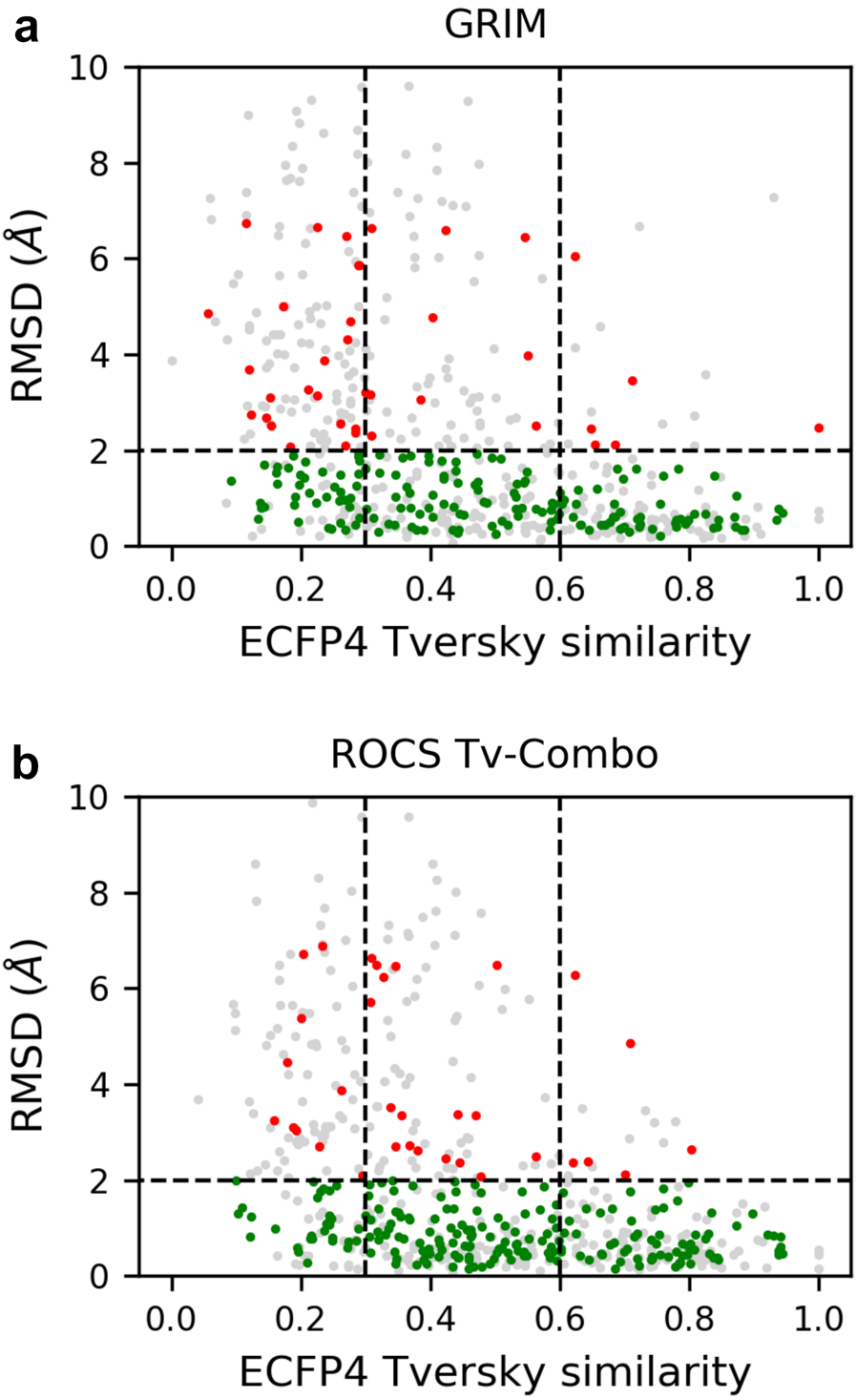
Rescoring performance versus chemical similarity between the fragment and the reference molecule. **a** GRIM. **b** ROCS Tv-combo. RMSD is computed between the predicted and native poses of a fragment. Chemical similarity between the docked fragment and the reference molecule is evaluated using ECFP4 Tversky similarity (α = 0.95 on the docking pose and β = 0.05 on the reference). Colors indicate whether rescoring improves (green), worsens (red) or has no effects on pose prediction (grey), as compared to ChemPLP and considering that docking is successful if RMSD < 2 Å)

Independent of the method, we observed that rescoring is more efficient if the reference molecules are fragments (Compare *F* and *D* in Fig. 4 and Additional file 1: Figure S2). The success rates obtained with the reference drug-like ligands are 5% to 15% lower. Combining the two reference sets yields the best performance of GRIM and ROCS, but not IFP. GRIM uses both fragment and drug-like ligand references to select the best docking solution (Fig. 5). A similar trend is found when using ROCS with the Tv-combo score while ROCS maximal Tc-combo score almost exclusively picks fragment references. Overall, the chemical similarity between the docked fragment and the reference molecule used to predict the best pose is slightly higher when using ROCS Tv-combo than when using GRIM (Fig. 6). GRIM especially picks a higher proportion of dissimilar references (35% vs. 25% of pairs with TvECFP4 < 0.3).

#### Reference molecules diversity

**Fig. 7:**
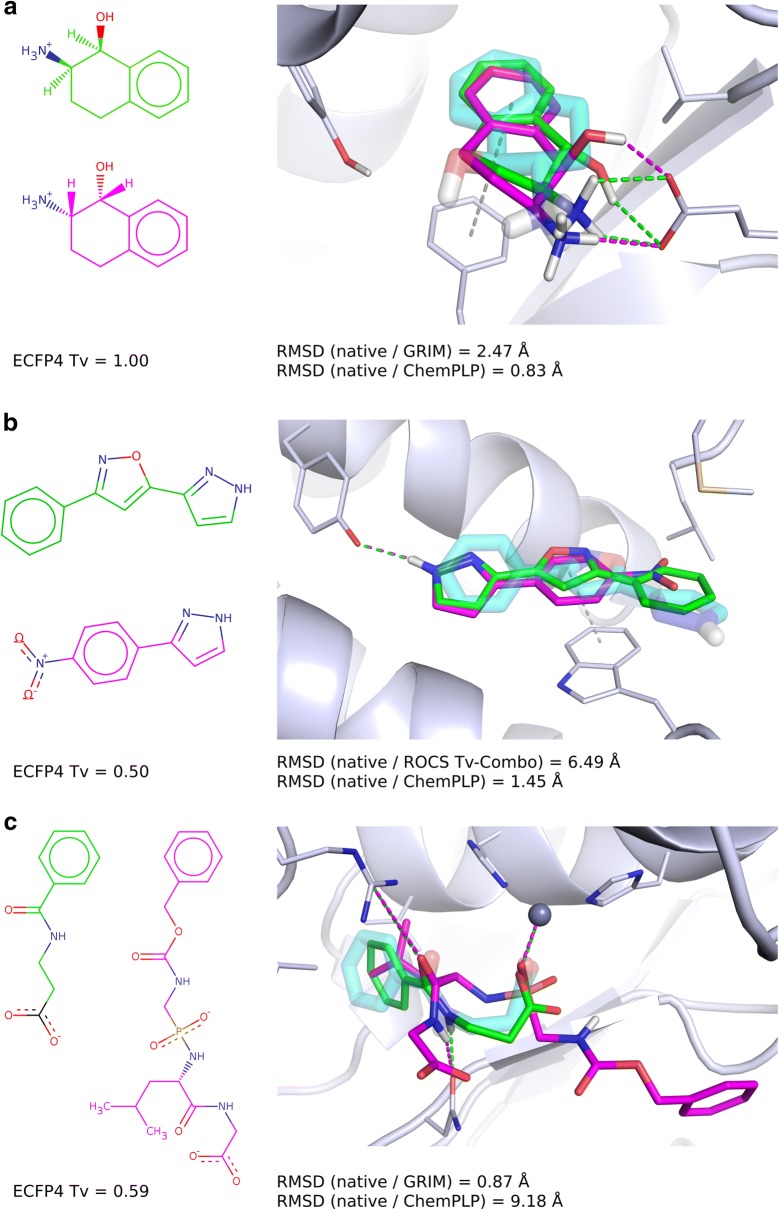
Examples of rescoring failures and successes. On the left: the docked fragment (green) and the reference molecule (magenta). On the right: comparison of the native pose (transparent cyan sticks), the pose selected by rescoring (green) and that of the corresponding reference (magenta). **a** Docking of 2-Amino-1,2,3,4-tetrahydronaphthalen-1-ol (HET ID: TTL, PDB ID: 2AN5) in the phenylethanolamine N-methyltransferase (P11086; PDB ID: 3KQT). GRIM rescoring uses a fragment reference (HET ID: CTL; PDB ID: 2AN3). **b** Docking of 3-phenyl-5-(1H-pyrazol-3-yl)isoxazole (HET ID: D25; PDB ID: 2VCQ) in the hematopoietic prostaglandin D synthase (O60760; PDB ID: 2VCZ). ROCS rescoring uses a fragment reference (HET ID: VC3; PDB ID: 2VCZ). **c** Docking of N-(phenylcarbonyl)-beta-alanine (HET ID: BYA; PDB ID: 3FGD) in the thermolysin (P00800; PDB ID: 4H57). GRIM rescoring uses a drug-like ligand reference (HET ID: UBT; PDB ID: 3T8G)

Are the reference molecules which are chemically similar to docked fragments more suitable for rescoring? Both GRIM and ROCS tend to select a correct pose when the similarity between the docked fragment and the picked reference molecule is high (TvECFP4 > 0.6, Fig. 6 and Additional file 1: Figure S4). However, we observed several rescoring failures. One example is the docking of 2-Amino-1,2,3,4-tetrahydronaphthalen-1-ol in phenylethanolamine N-methyltransferase. The pose selected by ChemPLP is more accurate than the one selected by GRIM (Fig. 7a). In both cases, the similarity between the fragment and reference molecule is maximal (TvECFP4 = 1.00), however, they are stereoisomers and therefore their 3D-structures do not superimpose [33]. Another example of a rescoring failure is the docking of 3-phenyl-5-(1H-pyrazol-3-yl)isoxazole in hematopoietic prostaglandin D synthase (Fig. 7b). The ROCS pose superimposes on the native pose, yet head to tail. The native pose shows only one directional interaction, an aromatic interaction between the fragment central isoxazole ring and a tryptophan. The reference molecule also stacks onto the tryptophan via its phenyl ring, and forms an additional directional interaction, a hydrogen bond between the pyrazole group and a tyrosine. The docked fragment contains a pyrazole group too, however it does not form a polar interaction with the protein.

The number of rescoring failures only slightly increases when the similarity between the docked fragment and the picked reference molecule decreases. But, most importantly, the number of rescoring successes is about twice higher than the number of rescoring failures when the picked reference structure and docked fragment are dissimilar (TvECFP4 < 0.3 on Fig. 6 and Additional file 1: Figure S4). In the example shown on Fig. 7c, the native and GRIM poses of the N-(phenylcarbonyl)-beta-alanine as well as that of the drug-like ligand UBTLN26 used as GRIM reference make the same polar interactions with the thermolysin binding site despite a limited overlap of the fragment and drug-like ligand atoms.

## Discussion

### IFP, GRIM or ROCS, what is the best choice?

In this benchmark exercise, we compared the performance of interaction fingerprints (IFP), interaction graphs (GRIM) and shape comparisons (ROCS). Statistics on the success rate in pose prediction suggest that the IFP method shows inferior performance than GRIM, which in turn is inferior to ROCS. This ranking coincides with the granularity of the encoding of the structural information used for the rescoring. IFP are based on the comparison of binding modes, but do not encode the geometry of interactions, nor their arrangement in space. In addition, the encoding per residue does not capture the number of interactions of the same type being established between the ligand and a protein residue. Like IFP, GRIM is based on the interactions between the ligand and the protein, but with a detailed encoding of their position and geometry. Moreover, GRIM is able to find the same motif  in two different binding modes while tolerating variations in the position of the protein atoms involved in the common interactions. ROCS uses only the information provided by the ligand atoms and therefore does not explicitly encode the interactions made with the protein. Nevertheless, the superposition of the pharmacophoric properties of the docked fragment and the reference molecule implies not only that interactions of the same type are formed, but also that the ligand atoms involved in these interactions occupy strictly the same position in the protein site.

The three rescoring approaches have different strengths and weaknesses, and are therefore not necessarily applicable in the same situations. IFP requires consistent numbering of residues in all the protein structures, precluding comparison of binding modes involving incomplete or mutated binding sites. However, IFP is the fastest of those three methods. Moreover, IFP also has the advantage of being able to find the interactions which are conserved when the protein undergoes important conformational changes. It is important to note that in the work described here, this situation does not occur. On the other hand, GRIM allows the comparison of any complexes, including mutated or even homologous proteins. Again, this has not been investigated here. ROCS is the most restrictive method since all the reference complexes have to be 3D-aligned onto the input protein structure before rescoring. The quality of the alignment determines the rescoring efficiency, so that the approach is more suitable for rigid sites.

### Rescoring success and protein-fragment complex properties

**Fig. 8:**
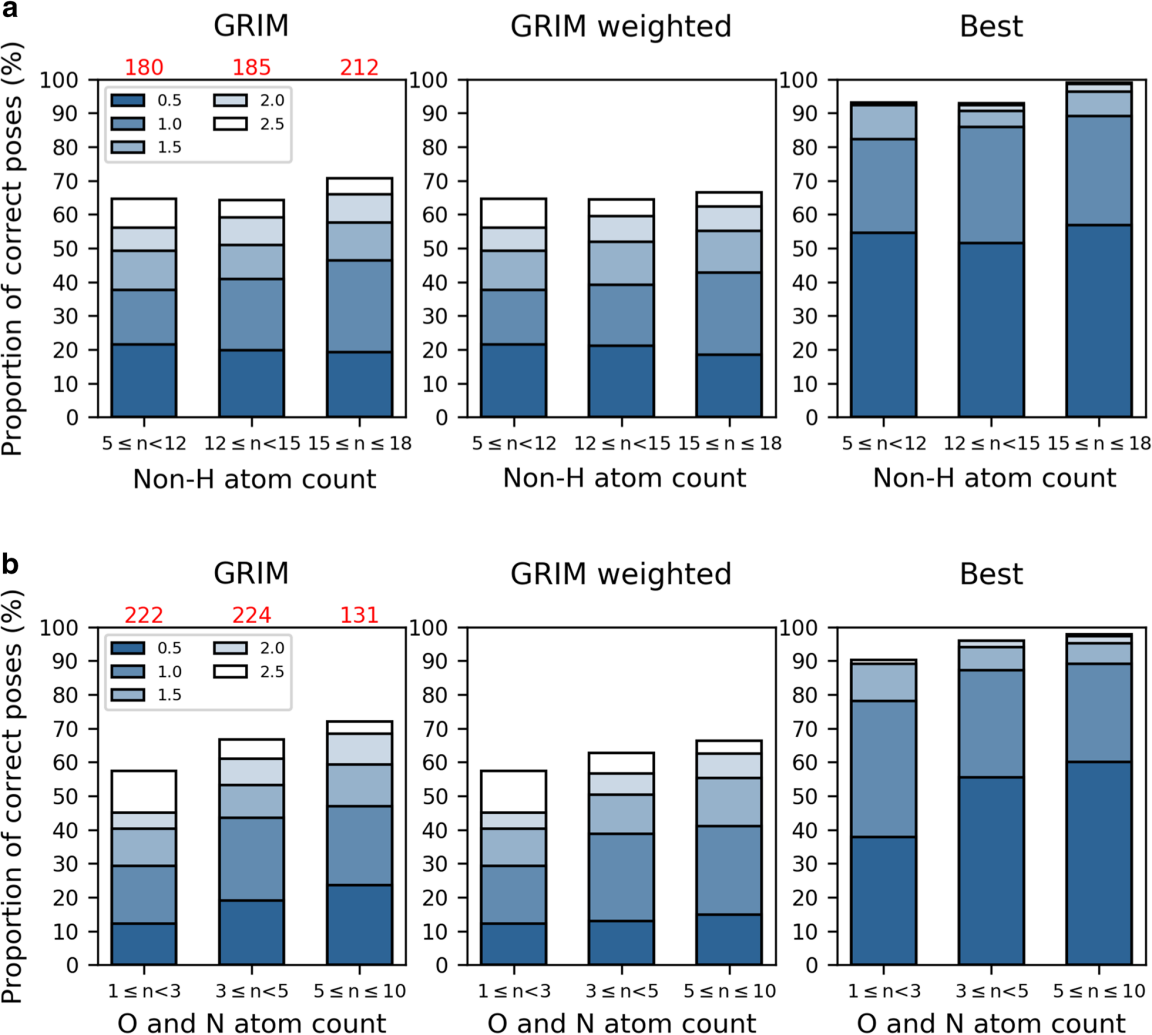
GRIM rescoring performance versus fragment properties. The reference molecules include both fragments and drug-like molecules (DF). Numbers in red indicate the number of fragments in the interval. The scoring performance is evaluated with the RMSD between the native and the docking poses (*GRIM*, left). This RMSD is corrected for the increase of the proportion of correct poses in the docking ensemble (*GRIM weighted*, center). This proportion is evaluated with the RMSD between the native pose and the best docking pose (*Best*, right). **a** Non-hydrogen atom count. **b** Oxygen and nitrogen atom count

Although IFP, GRIM as well as ROCS were able to recover the correct pose in a docking pose ensemble in many cases, rescoring failures also occurred. We thus wondered whether the rescoring performance depends on the physico-chemical properties of the fragment and protein binding site. Firstly, the performance of IFP, GRIM and ROCS are the same on the flexible and rigid binding sites. All the three methods are thus able to pick the correct pose in the appropriate protein conformation. The data also do not show a relationship between rescoring performance and the size of the binding site, or its surface polarity. By contrast, rescoring performance seems to change with fragment size. GRIM scoring accuracy increases when the fragment number of  non-hydrogen atoms increases but the variation is not significant (Fig. 8a, left panel and Additional file 1: Table S2A). In addition, this trend diminishes when the scoring accuracy is adjusted by considering docking accuracy (Fig. 8a, central panel), i.e., when correcting the increase of RMSD between the native and the docking poses for the increase of the proportion of correct poses in the docking ensemble (Fig. 8a, right panel). Focusing on the number of nitrogen and oxygen atoms which approximates the maximal number of hydrogen bonds the fragment can form with the protein site, we found that GRIM rescoring success rate is significantly lower if fragments contain only one or two nitrogen and/or oxygen atoms (Fig. 8b and Additional file 1: Table S2A). The proportion of correct poses is nearly one third smaller as compared to that of fragments containing three or more nitrogen and oxygen atoms. The same trend is observed using ROCS (Additional file 1: Figure S5 and Table S2B) but not using IFP, whose results seems to be independent of the fragment size (Additional file 1: Figure S6 and Table S2C).

### A high-quality benchmarking set for fragment docking

**Fig. 9:**
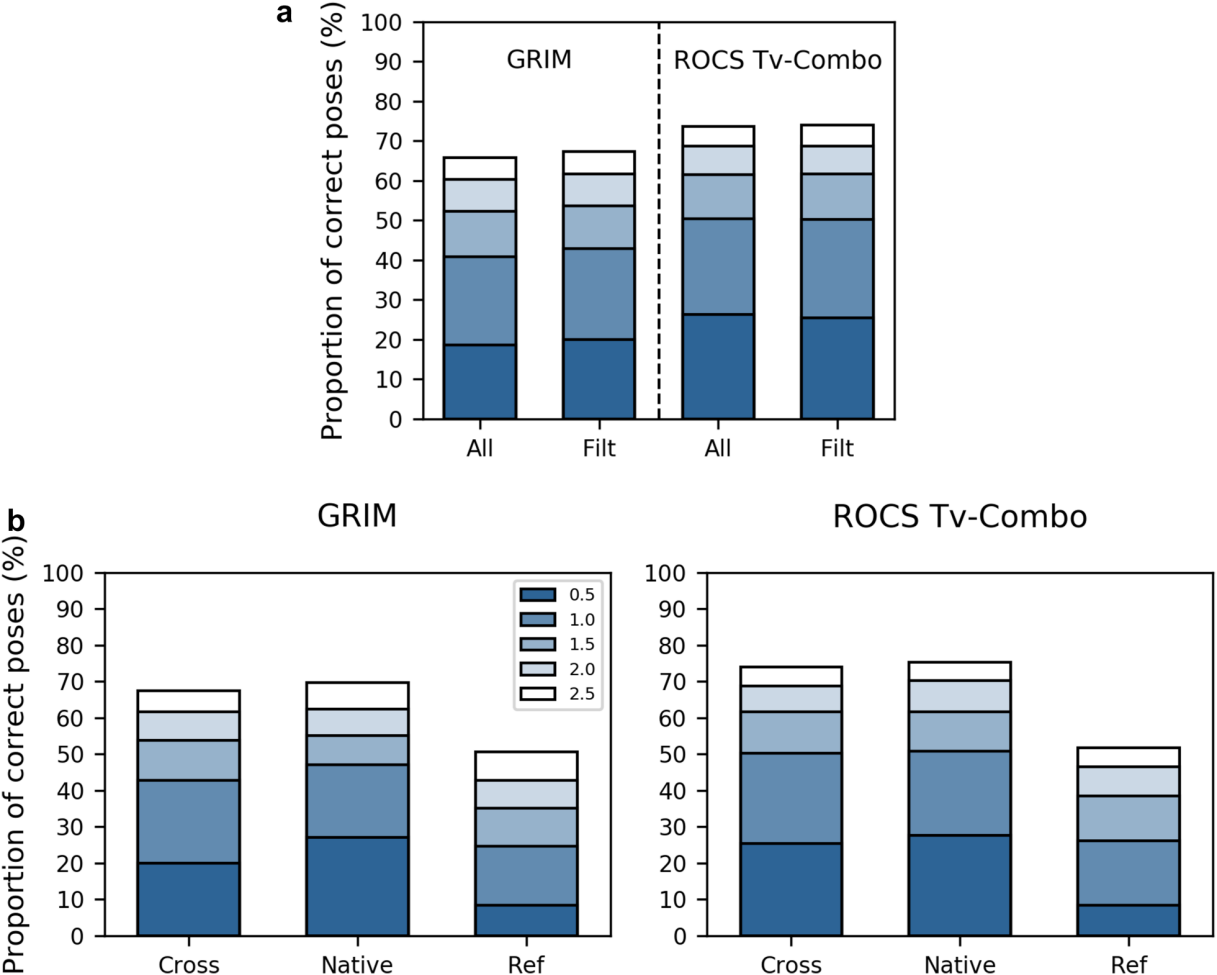
Variation of GRIM performance in pose prediction. The proportion of correct poses is based on the RMSD between the native and the docked poses of the fragment, considering five threshold values. Poses were selected by comparison to reference drug-like ligands and fragments (*DF*). **a** Filtering of low quality poses. Pose selection using the reference dataset before filtering with EDIA score (A*ll*, 832 docked fragments, 2082 reference molecules) and after filtering with EDIA score (*Filt*, 586 docked fragments, 1529 reference molecules). *Filt* is the dataset described in this study. **b** Native docking (*Native*) is compared to cross-docking using all the structures of the protein site (*Cross*) or only its representative structure (*Ref*)

Since the first comparisons of docking methods in the early 2000 [34, 35], benchmarking studies have regularly been published in the literature. Both pose prediction and virtual screening are generally discussed, with a focus on the scoring issue or on new developments such as flexible or covalent docking [36, 37]. Several studies have also aimed at providing guidelines for fair benchmarking, suggesting good practices in the design of benchmarking datasets and in data analysis [38–40]. Crystallographic structure quality is commonly accepted as an essential criterion, especially in pose prediction. Here, we verified the integrity of the fragment (or drug-like ligand) as well as any residues of its binding site. We validated the quality of the crystallographic structures by scoring the fit between electronic density and ligand structure. Noteworthy, GRIM and ROCS rescoring performance are hardly modified if low quality structures are not discarded from the reference dataset (Fig. 9a), suggesting that incomplete or approximate information on binding mode may be enough to guide pose selection.

The Astex diverse dataset [41] is a standard in pose prediction benchmarking. The 85 complexes between drug-like molecules and pharmaceutically relevant protein targets which constitute the dataset have been rigorously selected, and the match of atom coordinates and electron density was validated manually. In our dataset, which contains 30 times more structures, the structures of the native complexes of the docked fragments also passed quality filters, yet the match of atom coordinates and electron density has not been verified manually. The overlap between the two datasets is small. There are eleven common proteins, including five in complex with fragment (Additional file 1: Table S3). Similarly, the blind pose prediction cases proposed by CSAR and D3R challenges are different from those in our benchmarking dataset [19, 20, 42–45]. There are respectively only 27 and 7 common PDB entries (Additional file 1: Tables S4 and S5).

One strength of the present dataset lies in the multiple structures that are available for a protein, thereby allowing both the study of native docking and cross-docking. In native docking, or redocking, the input conformations of the ligand and the site come from the same PDB structure. Cross-docking uses input from different sources, and thus better reproduces conditions of prospective drug discovery investigations. Here, native docking of the fragment outperformed cross-docking considering a single protein structure (Fig. 9b), even if this structure is representative of the conformational ensemble and if the protein site is relatively rigid (Fig. 3c). Considering all the structures of protein brings the performance level of cross-docking back to that of native docking (Fig. 9b). Noteworthy, the standardization of inputs ensured that the protein structures are comparable, with identical residues in the binding site. Only amino acids and metal cofactors were included. Non-metal cofactor, other bound molecules and tightly bound water molecules were removed from structures. The absence of bound water in binding sites may be critical in docking [46]. We verified that it did not cause docking failures. In about half of the 32 cases where no correct poses were produced, interactions between the fragment and another ligand bound to the protein site were observed in the native complex.

### Fragments with multiple poses

Is a fragment binding pose unique? The development of the first approved fragment-based drug (vemurafenib) provides a first negative answer. The lead fragment 7-azaindole indeed showed multiple binding modes when crystallized in the ATP-binding site of the Pim-1 kinase [47]. Our recent analysis of the PDB identified about 100 fragments with multiple binding modes when considering a RMSD between two poses > 0.5 Å [21]. Low structural accuracy, conformational variation of fragments and changes in the protein environment in different crystal conditions explained many but not all examples of multiple binding modes, suggesting that more than a single pose may be relevant for fragment-based drug design [21].

**Fig. 10:**
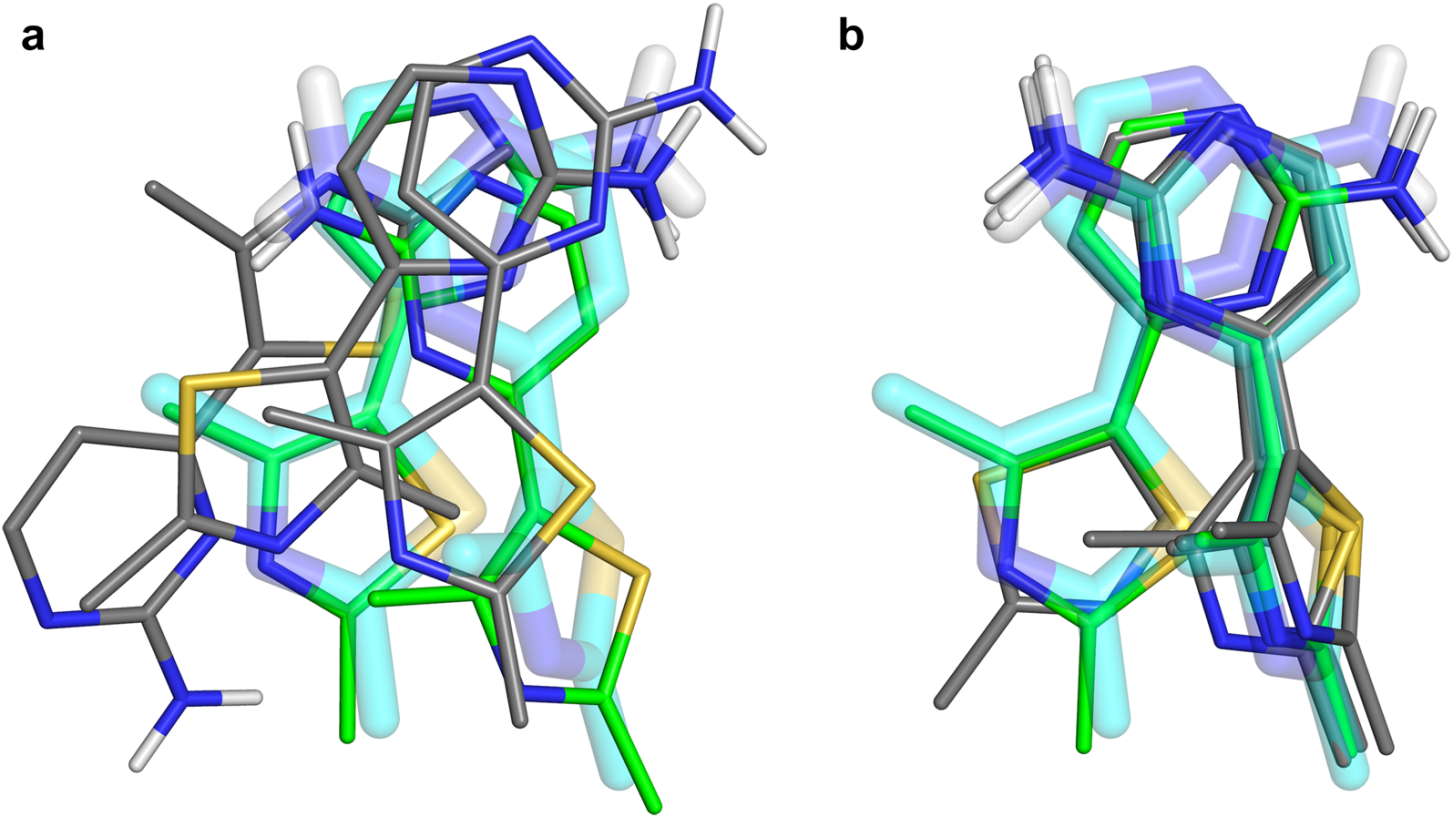
Multiple poses of CK2 within the cyclin-dependent kinase 2. Crystallographic structures revealed two binding modes in the protein site (Uniprot: P24941). The crystallographic poses are represented with transparent cyan sticks (PDB 1PXJ and 2C5O). In green are shown the top ranked correct poses (RMSD to the native pose < 1.0 Å). In grey are shown the top ranked incorrect poses. **a** The six best poses according to GRIM ranking. **b** The seven best poses according to ROCS ranking

In the docking benchmark set presented here, we distinguished the different native poses of a fragment by hierarchical clustering based on RMSD with a 1.0 Å cut-off. We identified only six fragments with multiple binding modes (Additional file 1: Table S6). We evaluated whether docking solutions comprise all the native poses, considering that a docking pose correctly predicts a native pose if the RMSD computed from their atomic coordinates is smaller than 1.0 Å. Unfortunately, the docking program failed to generate more than one correct pose for all but one fragment. Docking failures were most likely due to incorrect placement in absence of an organic cofactor, which were not included in the protein site. The only useful example is that of the CK2 fragment in the cyclin-dependent kinase 2 (CDK2). Many CDK2 structures are present in the dataset, and therefore, are used for docking, providing a total of 2040 CK2 poses. GRIM and ROCS placed solutions close to the two native poses among the six and seven top scored solutions, respectively (Fig. 10). In addition, ROCS ranked first the most representative native pose and proposed in the top of the list only solutions which are all close to the two native poses. By contrast, GRIM ranked only sixth the most representative native pose of CDK2 and suggested a various panel of binding modes (Fig. 10a). Noteworthy, the CK2 fragment was crystallized in the active and inactive forms of the protein, adopting two binding modes in the two protein conformations (PDB 2C50 and 1PXJ). The conformational changes in CDK2 are among the largest observed for all the proteins in the dataset (site RMSD Cα = 1.89 Å, site RMSD all = 2.71 Å). Remarkably, ROCS paired the “active” native pose with a site structure in the active form (2C5O and 3PXY, RMSD Cα = 0.73 Å) and the “inactive” native pose with a site structure in the inactive form (1PJX and 1H1R, RMSD Cα = 0.32 Å). By contrast, GRIM retrieved the two native poses in inactive forms of the site (PDB 2XNB and 1H1R).

## Conclusions

Interactions with the protein have already been considered in successful virtual screening campaigns. For example, hit rates of about 10% have been obtained in the search for human bromodomains inhibitors [48]. Here we demonstrated that binding mode information improves fragment pose prediction. Rescoring using the 3D-approaches GRIM and ROCS was more efficient than IFP rescoring based on 2D-fingerprints. Both fragments and drug-like ligands were suitable reference molecules. Importantly, GRIM and ROCS yielded successful rescoring when the docked fragment and reference molecules are structurally dissimilar. We also observed that rescoring performance tends to increase when the number of atoms, and more especially oxygen and nitrogen atoms, increases. A strong point common to all the three methods is the speed of calculation, which allows a large number of poses to be processed. We exploited this advantage to rank the poses obtained for docking a fragment in multiple conformations of the target protein site.

## Additional file

**Additional file 1. Figure S1.** Effect of the fragment input conformation on PLANTS performance; **Figure S2.** IFP, GRIM and ROCS performance in scenario 1 and scenario 3; **Figure S3.** Comparing Tanimoto and Tversky similarities for rescoring using IFP and ROCS; **Figure S4.** Chemical similarity between the docked fragment and the reference molecule picked by GRIM or ROCS Tv-Combo; **Figure S5.** ROCS Tv-Combo rescoring performance versus fragment properties; **Figure S6.** Tc-IFP rescoring performance versus fragment properties; **Table S1.** Description of the proteins in the benchmark set; **Table S2.** Kolmogorov-Smirnov test on non-weighted RMSD distributions binned by fragment properties; **Table S3.** Intersect with the Astex diverse dataset; **Table S4.** Intersect with D3R datasets; **Table S5.** Intersect with the CSAR datasets; **Table S6.** Fragments with multiple crystallographic poses.

## Abbreviations

CDK2: cyclin-dependent kinase 2
D3R: Drug Design Data Resource
GRIM: graph matching of interaction patterns
IPA: interaction pseudo atoms
HTS: high throughput screening
IFP: interaction fingerprints
MW: molecular weight
PDB: Protein Data Bank
RMSD: root mean square deviation
ROCS: rapid overlay of chemical structures
