# Supplementary material for: Binding mode information improves fragment docking
Source: J Cheminform. 2019 Mar 22;11:24. doi: 10.1186/s13321-019-0346-7 (PMC6431075; doi:10.1186/s13321-019-0346-7)
Supplement: Supplementary file 1 — Additional file 1. Figure S1. Effect of the fragment input conformation on PLANTS performance; Figure S2. IFP, GRIM and ROCS performance in scenario 1 and scenario 3; Figure S3. Comparing Tanimoto and Tversky similarities for rescoring using IFP and ROCS; Figure S4. Chemical similarity between the docked fragment and the reference molecule picked by GRIM or ROCS Tv-Combo; Figure S5. ROCS Tv-Combo rescoring performance versus fragment properties; Figure S6. Tc-IFP rescoring performance versus fragment properties; Table S1. Description of the proteins in the benchmark set; Table S2. Kolmogorov-Smirnov test on non-weighted RMSD distributions binned by fragment properties; Table S3. Intersect with the Astex diverse dataset; Table S4. Intersect with D3R datasets; Table S5. Intersect with the CSAR datasets; Table S6. Fragments with multiple crystallographic poses. [file 13321_2019_346_MOESM1_ESM.pdf]

Supplementary Material

Binding mode information improves fragment docking

Célien Jacquemard[1], Malgorzata N. Drwal[1], Jérémy Desaphy[2], Esther Kellenberger[1]

1 Laboratoire d'innovation thérapeutique, UMR7200 CNRS Université de Strasbourg, 67400 Illkirch, France.

2 Lilly Research Laboratories, Eli Lilly and Company, Indianapolis, IN 46285, USA.

Table of contents

Figure S1 – Effect of the fragment input conformation on PLANTS performance. 2

Figure S2 – IFP, GRIM and ROCS performance in scenario 1 and scenario 3. 3

Figure S3 – Comparing Tanimoto and Tversky similarities for rescoring using IFP and ROCS. 4

Figure S4 – Chemical similarity between the docked fragment and the reference molecule picked by GRIM or ROCS Tv-Combo. 5

Figure S5 – ROCS Tv-Combo rescoring performance versus fragment properties. 6

Figure S6 – Tc-IFP rescoring performance versus fragment properties. 7

Table S1 – Description of the proteins in the benchmark set 8

Table S2 – Kolmogorov-Smirnov test on non-weighted RMSD distributions binned by fragment properties. 12

Table S3 – Intersect with the Astex diverse dataset 13

Table S4 – Intersect with the D3R datasets 14

Table S5 – Intersect with the CSAR datasets 15

Table S6 – Fragments with multiple crystallographic poses. 16

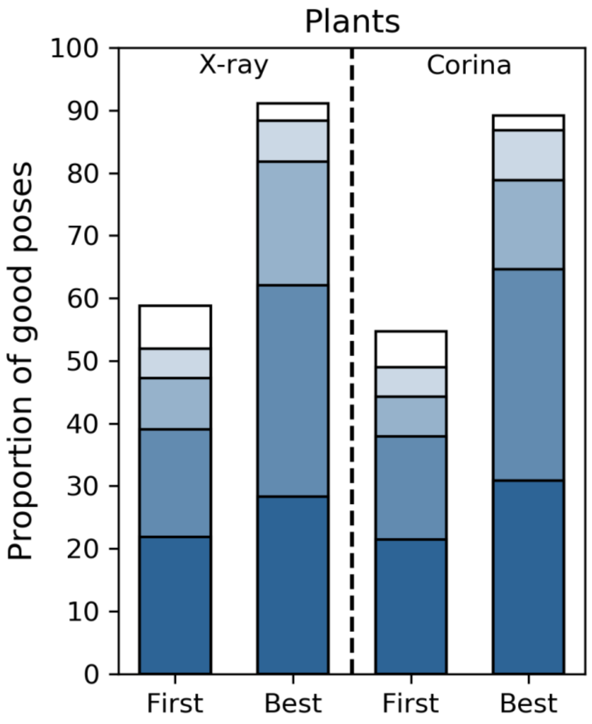

Figure S1 – Effect of the fragment input conformation on PLANTS performance.

The proportion of correct poses is based on the RMSD between the native and docked poses of the fragment, considering five threshold values (darker to brighter: 0.5, 1.0, 1.5, 2.0 and 2.5 Å). Proportions are calculated by considering a single pose within the ensemble generated for a complex, as follows: *First* denotes the top scored pose; *Best* denotes the closest to the native pose. The left panel (X-ray) shows results obtained when docking the crystallographic conformation of the fragment. The right part (Corina) shows results obtained when docking the modelled conformation, generated *ab initio* with Corina from the SMILES string.

More details about the modelling of 3D-conformation:

The X-ray structures of the fragments were converted into canonical SMILES using OpenEye Python API 2017, ignoring aromaticity and preserving stereochemistry. The 3D-structures were generated using CORINA v3.40 corina (options: write formal charge, write all hydrogen atoms, preserve stereochemistry, explore alternative conformations of rings).

A

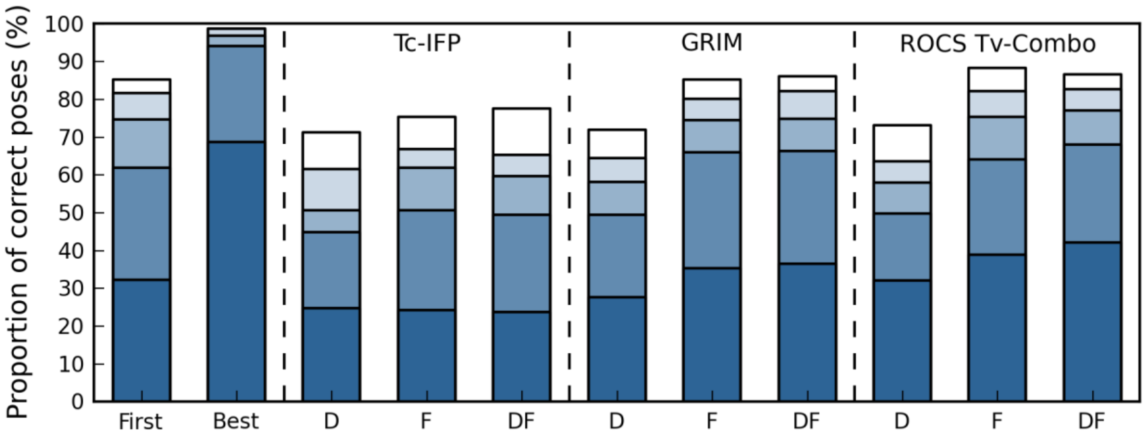

B

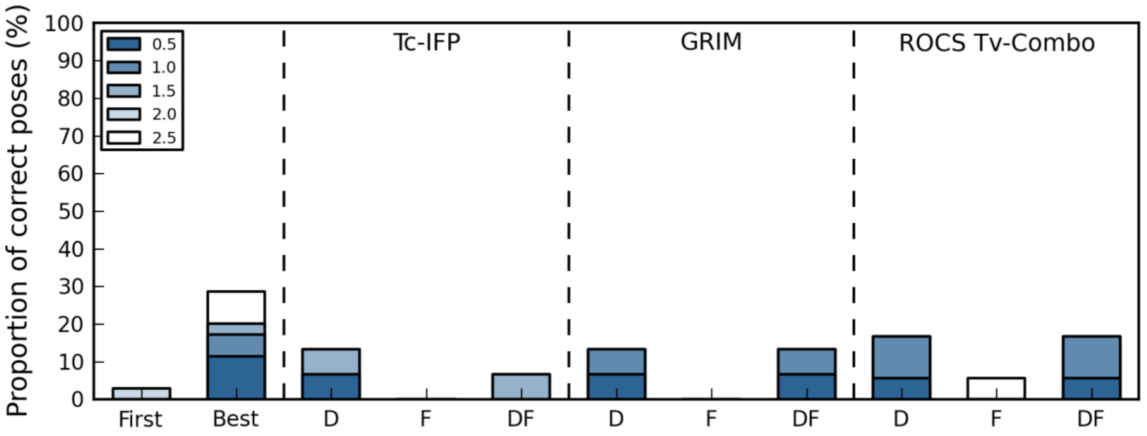

Figure S2 – IFP, GRIM and ROCS performance in scenario 1 and scenario 3.

The proportion of correct predictions is based on the RMSD between the predicted and native poses of fragment, considering five threshold values. Proportions are calculated by considering a single pose within the ensemble generated for a complex, as follows: *First* denotes the top scored pose; *Best* denotes the closest to the native pose; *D*, *F* and *DF* denote the poses selected by comparison to, respectively, reference drug-like ligand, reference fragments and both. **(A)** The 24 protein sites without major scoring issues (scenario 1). **(B)** The five protein sites with severe pose generation issues (scenario 2).

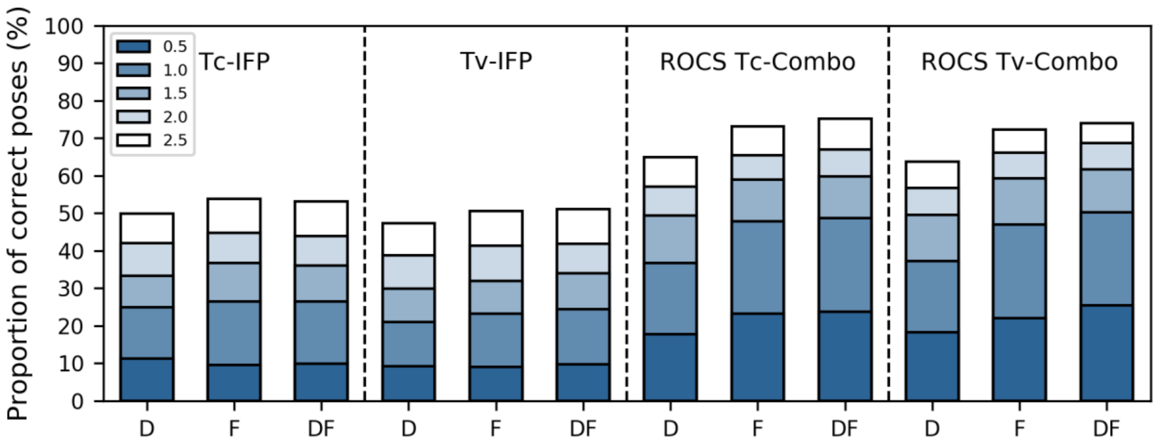

Figure S3 – Comparing Tanimoto and Tversky similarities for rescoring using IFP and ROCS.

The proportion of correct poses is based on the RMSD between the native and docked poses of the fragment, considering five threshold values. IFP and ROCS poses were selected by comparison to three sets of reference molecules: drug-like ligands (*D*), fragments (*F*) and both (*DF*).

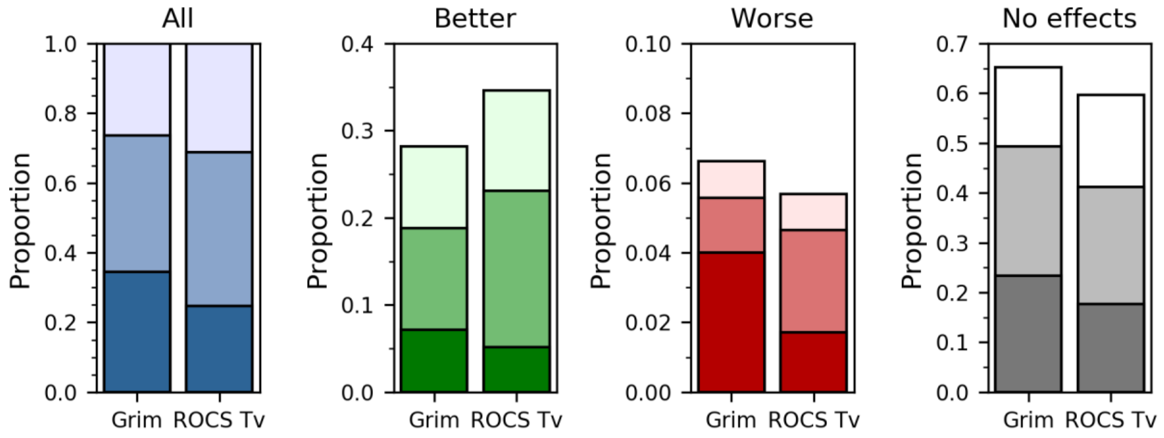

Figure S4 – Chemical similarity between the docked fragment and the reference molecule picked by GRIM or ROCS Tv-Combo.

Proportion of pairs with a TvECFP4 similarity < 0.3 (dark), ≥ 0.3 and < 0.6 (medium) and ≥ 0.6 (light) is given for all the 586 dockings (*All*, in blue), only successful rescoring cases (*Better*, in green), only rescoring failures (*Worse*, in red) or only cases where rescoring has no effects on pose prediction (*No effects*, in grey). Rescoring success corresponds to a decrease of the RMSD to the native pose from ≥ 2.0 Å to < 2 Å. Rescoring failure corresponds to an increase of the RMSD to the native pose from < 2 Å to ≥ 2.0 Å.

A

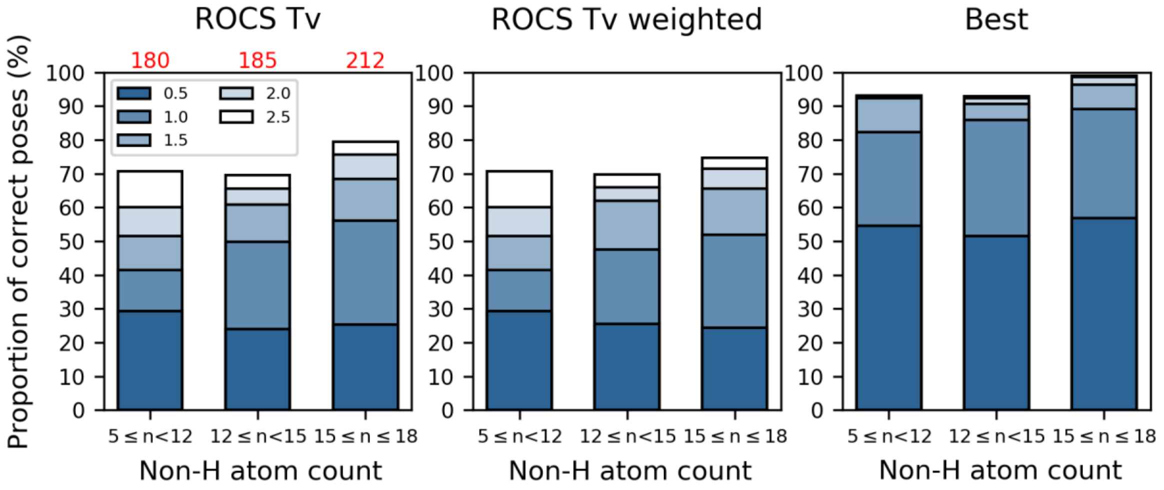

B

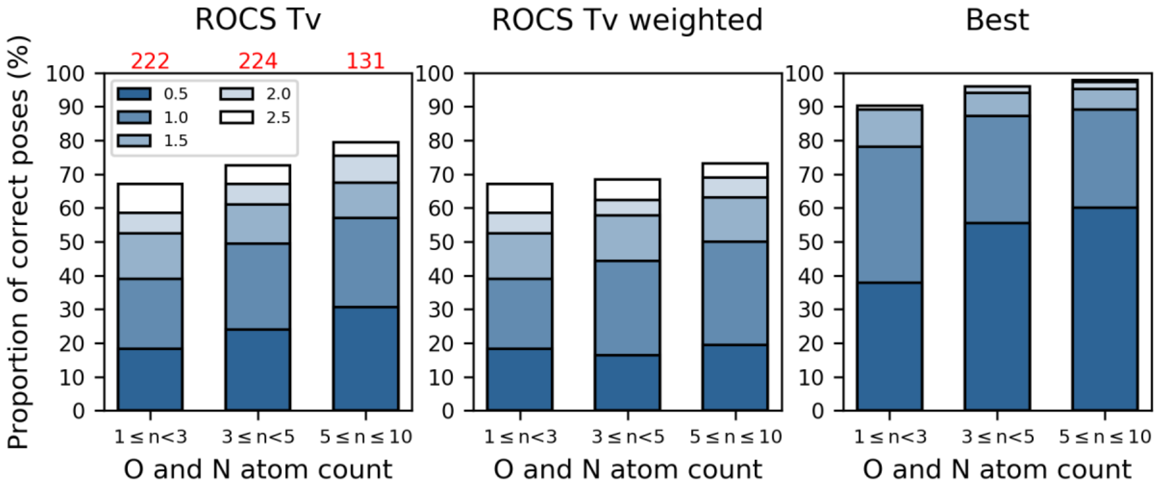

Figure S5 – ROCS Tv-Combo rescoring performance versus fragment properties.

The reference molecules include both fragments and drug-like molecules (*DF*). Numbers in red indicate the number of fragments in the interval. The scoring performance is evaluated with the RMSD between the native and docked poses (*ROCS Tv*, left). This RMSD is corrected for the increase of the proportion of correct poses in the docking ensemble (*ROCS Tv weighted*, center). This proportion is evaluated with the RMSD between the native pose and the best docking pose (*Best*, right). **(A)** Non-hydrogen atom count. **(B)** Oxygen and nitrogen atom count**.**

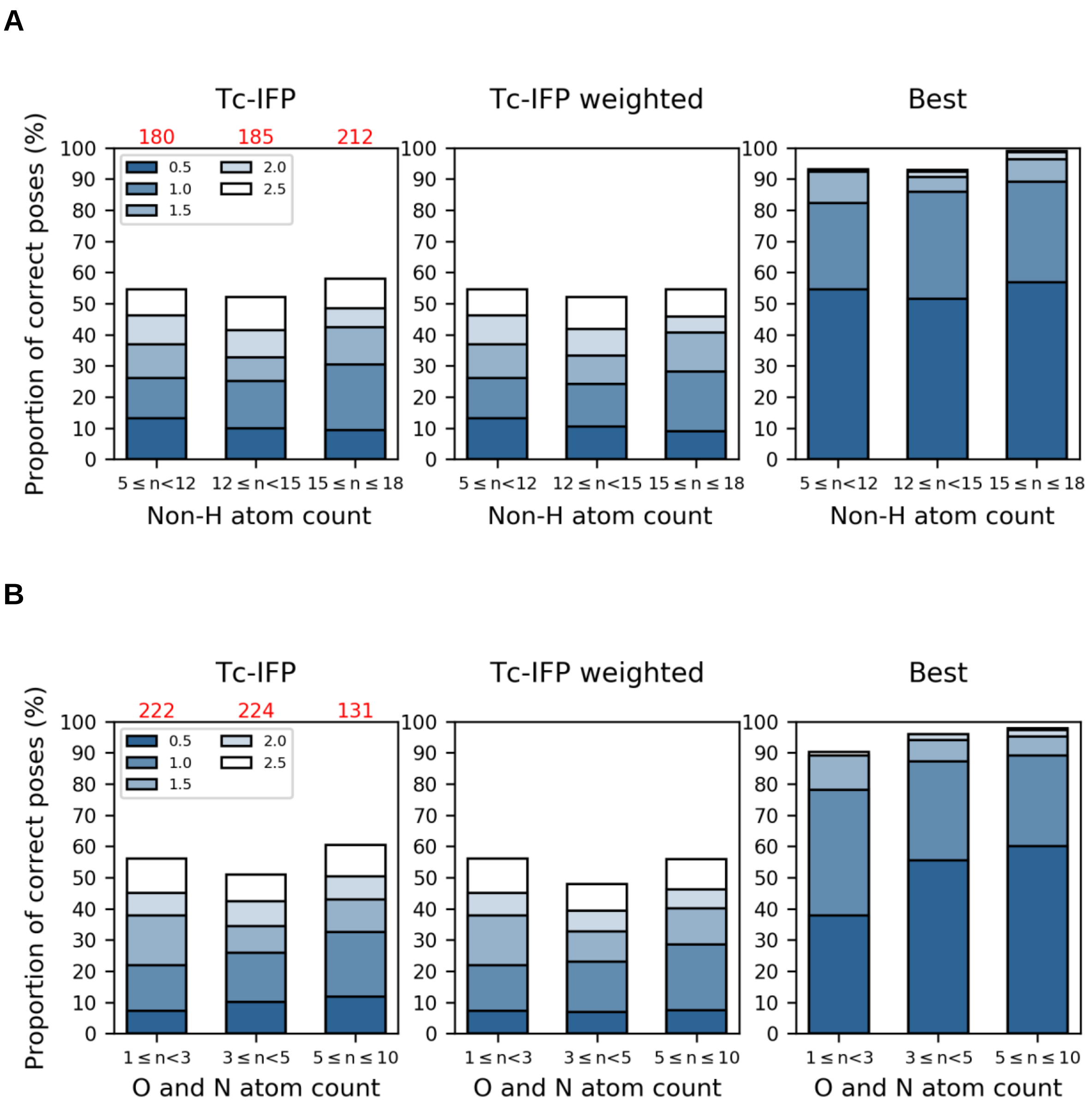

Figure S6 – Tc-IFP rescoring performance versus fragment properties.

The reference molecules include both fragments and drug-like molecules (*DF*). Numbers in red indicate the number of fragments in the interval. The scoring performance is evaluated with the RMSD between the native and docked poses (*Tc-IFP*, left). This RMSD is corrected for the increase of the proportion of correct poses in the docking ensemble (*Tc-IFP weighted*, center). This proportion is evaluated with the RMSD between the native pose and the best docking pose (*Best*, right). **(A)** Non-hydrogen atom count. **(B)** Oxygen and nitrogen atom count**.**

Table S1 – Description of the proteins in the benchmark set

Uniprot ID	Uniprot AC	Protein class	Scoring issue	Number of fragment HET codes	Number of ligand HET codes	Number of fragment structures	Number of ligand structures	Cα mean RMSD (Å)	All atom mean RMSD (Å)	Binding site residues	Mean cavity volume (Å^3)	Binding site hydrophobicity (%)	Ions in binding site
ALBU_HUMAN	P02768	Miscellaneous	Yes	2	12	3	19	2,18	2,39	75	893,6	46,8	
PYGM_RABIT	P00489	Transferase	No	4	6	4	7	0,28	0,66	55	486,7	25,7	
ALDR_HUMAN	P15121	Oxidoreductase	Yes	7	16	7	34	0,33	0,56	36	474,6	16,9	
INHA_MYCTU	P9WGR1	Oxidoreductase	No	3	8	5	12	1,31	1,65	43	645,9	50,4	
AOFB_HUMAN	P27338	Oxidoreductase	Yes	4	4	16	7	0,28	0,50	41	443,7	31,5	
BRD4_HUMAN	O60885	Chromatin regulator	Yes	13	13	13	22	0,34	0,63	25	388,3	51,8	
ESR2_HUMAN	Q92731	Activator	Yes	3	3	6	5	0,18	0,61	43	305,2	66,5	
COBT_SALTY	Q05603	Transferase	No	8	7	14	13	0,13	0,31	42	412,1	47,1	
DNLJ_ENTFA	Q837V6	Ligase	No	3	2	3	2	0,79	1,27	39	706,7	32,6	
GRIA2_RAT	P19491	Ion channel	No	21	2	53	9	1,13	1,27	51	231,1	36,1	
KHK_HUMAN	P50053	Transferase	No	3	4	5	4	1,48	1,74	33	420,0	58,1	

PIN1_HUMAN	Q13526	Isomerase	No	8	3	9	3	0,28	0,98	32	274,3	30,7	
Q9HV14_PSEAE	Q9HV14	Miscellaneous	No	3	3	3	3	0,18	0,39	51	454,1	39,6	
Q4Q5S8_LEIMA	Q4Q5S8	Transferase	Yes	4	6	4	6	0,58	0,82	38	700,0	25,7	
MAP1_ECOLI	P0AE18	Hydrolase	No	15	4	17	4	0,36	0,58	29	380,9	10,0	
NOSO_BACSU	O34453	Oxidoreductase	Yes	2	13	2	13	0,19	0,72	30	877,0	25,7	
HS90A_HUMAN	P07900	Chaperone	Yes	42	64	57	85	1,45	1,77	48	540,8	42,8	
PDE4D_HUMAN	Q08499	Hydrolase	Yes	3	13	8	48	0,27	0,49	51	648,8	31,9	Zn Mg
Q95WL3_TRYCR	Q95WL3	Transferase	No	5	3	12	3	0,28	0,66	31	318,1	18,9	Mg Mg Mg
AMP1_PLAFQ	O96935	Hydrolase	No	3	4	3	4	0,17	0,36	39	551,4	33,9	Zn
PIM1_HUMAN	P11309	Transferase	Yes	16	31	16	32	0,31	0,63	42	510,3	53,5	
ADRB1_MELGA	P07700	Receptor	No	3	3	6	8	0,23	0,39	78	551,6	32,4	
PK3CG_HUMAN	P48736	Transferase	Yes	3	35	3	35	0,77	1,40	43	627,6	46,3	
PDE10_HUMAN	Q9Y233	Hydrolase	Yes	22	14	24	25	0,28	0,46	44	555,3	34,4	Mg
HPGDS_HUMAN	O60760	Isomerase	Yes	4	8	7	14	0,47	0,75	38	333,5	44,8	Mg
BACE1_HUMAN	P56817	Hydrolase	Yes	16	136	19	208	0,86	1,27	60	739,0	26,2	
DYR1A_HUMAN	Q13627	Transferase	Yes	7	3	17	13	0,42	0,71	37	546,3	45,9	
RORG_HUMAN	P51449	Receptor	Yes	6	4	6	5	0,31	0,61	53	770,4	49,2	
GRIK1_RAT	P22756	Ion channel	No	3	9	17	20	0,38	0,66	37	363,2	27,4	

ACES_TETCF	P04058	Hydrolase	Yes	6	9	15	10	0,21	0,48	60	658,5	19,1	
THER_BACTH	P00800	Hydrolase	Yes	11	5	12	5	0,38	0,63	29	229,2	34,0	Zn
ESR1_HUMAN	P03372	Activator	No	11	18	18	24	0,44	0,82	48	416,9	63,1	
PGH1_SHEEP	P05979	Oxidoreductase	No	4	5	12	7	0,21	0,45	51	409,1	64,5	
PYRD_TRYCC	Q4D3W2	Oxidoreductase	No	10	6	20	11	0,42	0,59	29	434,8	25,3	
LKHA4_HUMAN	P09960	Hydrolase	Yes	11	10	12	14	0,15	0,29	57	575,4	24,6	Zn
PA2B8_DABRR	P59071	Toxin	No	2	1	2	1	0,16	0,24	46	352,2	41,9	
CSK21_HUMAN	P68400	Transferase	No	6	10	7	13	1,12	1,58	39	499,5	49,8	
PTN1_HUMAN	P18031	Hydrolase	No	3	4	3	4	0,17	0,45	40	204,6	32,8	
PGH2_MOUSE	Q05769	Oxidoreductase	No	6	7	18	25	0,20	0,50	46	500,5	61,0	
DAPK1_HUMAN	P53355	Transferase	Yes	3	1	3	1	0,52	0,80	43	460,7	56,2	
CHK1_HUMAN	O14757	Transferase	No	5	24	5	25	0,48	0,75	46	466,8	50,4	
TGT_ZYMMO	P28720	Transferase	No	14	6	19	7	0,61	0,89	45	397,4	37,2	
FPPS_HUMAN	P14324	Transferase	No	7	5	16	9	0,19	0,57	37	381,6	32,7	Mg Mg Mg
Q9BJJ9_PLAFA	Q9BJJ9	Miscellaneous	Yes	5	5	11	8	0,27	0,74	36	613,3	45,7	
PANC_MYCTU	P9WIL5	Ligase	Yes	5	5	10	6	0,25	0,63	48	N/A	N/A	
CP121_MYCTU	P9WPP7	Oxidoreductase	No	7	6	8	10	0,18	0,22	45	1036,5	44,6	
D0VX11_9SAUR	D0VX11	Hydrolase	No	4	3	5	4	0,46	0,78	40	507,2	41,9	

MMP12_HUMAN	P39900	Hydrolase	Yes	6	15	6	23	0,23	0,46	35	398,4	36,3	Zn
HYES_HUMAN	P34913	Hydrolase	Yes	21	6	25	8	0,61	1,01	64	804,0	43,6	
CDK2_HUMAN	P24941	Transferase	Yes	38	118	44	152	1,07	1,62	48	539,6	45,2	
TNKS2_HUMAN	Q9H2K2	Transferase	No	26	13	72	26	0,96	1,62	45	455,1	36,4	
CAH2_HUMAN	P00918	Lyase	Yes	110	45	122	45	0,20	0,49	37	326,7	27,4	Zn
CPXA_PSEPU	P00183	Oxidoreductase	Yes	3	3	68	3	0,84	1,12	40	321,4	58,0	
PDPK1_HUMAN	O15530	Transferase	Yes	6	21	6	25	0,41	0,94	49	513,9	47,7	Mg
BLA1_STEMA	P52700	Hydrolase	Yes	6	4	7	6	0,23	0,92	27	225,3	39,4	Zn Zn
AMPN_ECOLI	P04825	Hydrolase	No	5	3	5	4	0,21	0,73	37	545,8	23,0	Zn
JAK2_HUMAN	O60674	Transferase	Yes	5	14	6	25	0,82	1,13	44	595,5	47,7	
AMPC_ECOLI	P00811	Hydrolase	Yes	16	3	30	3	0,83	1,15	36	410,0	27,8	
PNMT_HUMAN	P11086	Transferase	Yes	29	3	57	5	0,24	0,40	42	185,4	27,4	
Q9WYE2_THEMA	Q9WYE2	Miscellaneous	No	4	4	8	7	0,47	0,68	36	427,5	34,8	
ACCC_ECOLI	P24182	Ligase	Yes	6	5	11	9	0,93	1,11	44	493,9	39,2	
GSK3B_HUMAN	P49841	Signal transduction inhibitor	Yes	2	16	4	28	0,80	1,07	39	538,5	45,6	
NOS2_MOUSE	P29477	Oxidoreductase	Yes	7	7	12	14	0,26	0,50	37	564,7	22,5	
BGLA_THEMA	Q08638	Hydrolase	No	11	5	22	15	0,17	0,44	34	481,8	16,3	

Table S2 – Kolmogorov-Smirnov test on non-weighted RMSD distributions binned by fragment properties.

Goodness of fit is evaluated using *D* followed by two-tailed *p-value*. Failed tests are indicated in red ($\alpha = 0.05$). **(A)** GRIM. **(B)** ROCS Tv-Combo. **(C)** Tc-IFP. Distributions are not normal (see Figures 9, S5 and S6).

A

**GRIM**		Non-hydrogen atom count		
	Interval	1	2	3
Oxygen and nitrogen count	1		0.06685, 0.89	0.1123, 0.20
	2	0.1770, 0.034		0.09382, 0.30
	3	0.2374, 0.0018	0.08121, 0.38	

B

**ROCS Tv-Combo**		Non-hydrogen atom count		
	Interval	1	2	3
Oxygen and nitrogen count	1		0.1097, 0.32	0.1974, 0.0017
	2	0.1286, 0.23		0.1056, 0.18
	3	0.1962, 0.016	0.1176, 0.062	

C

**Tc-IFP**		Non-hydrogen atom count		
	Interval	1	2	3
Oxygen and nitrogen count	1		0.1216, 0.21	0.1093, 0.23
	2	0.08920, 0.68		0.1037, 0.19
	3	0.1055, 0.49	0.1008, 0.1546	

Table S3 – Intersect with the Astex diverse dataset

**Protein (UniProt ID/AC)**	**PDB ID**	**HET ID**	**Class**
PNMT_HUMAN/P11086	1HNN	SKF	Fragment
AMPC_ECOLI/P00811	1L2S	STC	Drug-like ligand
PANC_MYCTU/P9WIL5	1N2J	PAF	Fragment
TGT_ZYMMO/P28720	1N2V	BDI	Fragment
CPXA_PSEPU/P00183	1P2Y	NCT	Fragment
PGH1_SHEEP/P05979	1Q4G	BFL	Fragment
ESR1_HUMAN/P03372	1SJ0	E4D	Drug-like ligand
ALDR_HUMAN/P15121	1T40	ID5	Drug-like ligand
PDE4D_HUMAN/Q08499	1XOQ	ROF	Drug-like ligand
CHK1_HUMAN/O14757	2BR1	PFP	Drug-like ligand
HS90A_HUMAN/P07900	2BSM	BSM	Drug-like ligand

Table S4 – Intersect with the D3R datasets

Protein (UniProt ID/AC)	PDB ID	HET ID	Class
HS90A_HUMAN/P07900	3OW6	MEX	Fragment
HS90A_HUMAN/P07900	3OWD	MEY	Drug-like ligand
HS90A_HUMAN/P07900	4YKU	4ER	Fragment
HS90A_HUMAN/P07900	4YKW	4ES	Fragment
HS90A_HUMAN/P07900	4YKX	4ET	Fragment
HS90A_HUMAN/P07900	4YKY	4EU	Fragment
HS90A_HUMAN/P07900	4YKZ	4EV	Fragment

Table S5 – Intersect with the CSAR datasets

Protein (UniProt ID/AC)	PDB ID	HET ID	Class
ALBU_HUMAN/P02768	1HA2	SWF	Drug-like ligand
AMPC_ECOLI/P00811	2HDQ	C21	Fragment
AMPC_ECOLI/P00811	2HDR	4A3	Fragment
BACE1_HUMAN/P56817	2VKM	BSD	Drug-like ligand
BGLA_THEMA/Q08638	2J78	GOX	Fragment
BLA1_STEMA/P52700	2QDT	I38	Fragment
CAH2_HUMAN/P00918	2H15	B19	Drug-like ligand
CDK2_HUMAN/P24941	1PXP	CK8	Drug-like ligand
CDK2_HUMAN/P24941	2C5O	CK2	Fragment
CDK2_HUMAN/P24941	2FVD	LIA	Drug-like ligand
CHK1_HUMAN/O14757	4FT3	H1K	Drug-like ligand
CHK1_HUMAN/O14757	4FTU	7HK	Drug-like ligand
CHK1_HUMAN/O14757	4GH2	HK0	Drug-like ligand
ESR2_HUMAN/Q92731	2JJ3	JJ3	Drug-like ligand
ESR2_HUMAN/Q92731	2Z4B	DC8	Drug-like ligand
GRIA2_RAT/P19491	1SYH	CPW	Fragment
HS90A_HUMAN/P07900	1YC1	4BC	Drug-like ligand
HS90A_HUMAN/P07900	3EKO	PYU	Fragment
MAP1_ECOLI/P0AE18	2P98	YE7	Fragment
PIM1_HUMAN/P11309	3BGZ	VX3	Drug-like ligand
PNMT_HUMAN/P11086	1HNN	SKF	Fragment
PTN1_HUMAN/P18031	2AZR	982	Fragment
PTN1_HUMAN/P18031	2B07	598	Drug-like ligand
PTN1_HUMAN/P18031	2QBR	910	Drug-like ligand
PTN1_HUMAN/P18031	2QBS	024	Drug-like ligand
TGT_ZYMMO/P28720	1ENU	APZ	Fragment
TGT_ZYMMO/P28720	1S38	MAQ	Fragment

Table S6 – Fragments with multiple crystallographic poses.

**Uniprot ID**	**HET code**	**Number of**			**Remark** (c)
		**X-ray structures**	**X-ray poses** (a)	**Correct docked poses** (b)	
TNKS2_HUMAN	33E	6	3	1	Missing cofactor
Q95WL3_TRYCR	IPE	2	2	0	Missing cofactor
AMPC_ECOLI	F12	2	2	1	Missing cofactor
CPXA_PSEPU	CAM	2	2	0	Missing cofactor
CDK2_HUMAN	CK2	2	2	2	
PDE4D_HUMAN	IBM	2	2	0	Missing cofactor

(a) The number of crystallographic (X-ray) poses was evaluated as the number of clusters obtained using a RMSD threshold of 1.0 Å (see methods for more details).
(b) The RMSD of the coordinates of the crystallographic and docked poses is lower than 1.0 Å.
(c) Difficulties encountered during pose placement.
